# The ATPase Inhibitory Factor 1 is a Tissue-Specific Physiological Regulator of the Structure and Function of Mitochondrial ATP Synthase: A Closer Look Into Neuronal Function

**DOI:** 10.3389/fphys.2022.868820

**Published:** 2022-03-18

**Authors:** Sonia Domínguez-Zorita, Inés Romero-Carramiñana, José M. Cuezva, Pau B. Esparza-Moltó

**Affiliations:** ^1^ Departamento de Biología Molecular, Centro de Biología Molecular Severo Ochoa, (CSIC-UAM), Centro de Investigación Biomédica en Red de Enfermedades Raras (CIBERER) ISCIII, Instituto de Investigación Hospital 12 de Octubre, Universidad Autónoma de Madrid, Madrid, Spain; ^2^ Salk Institute for Biological Studies, La Jolla, CA, United States

**Keywords:** ATP synthase, ATPase inhibitory factor 1, neurons, oxidative phosphorylation, Mitohormesis, cellular signaling, reactive oxygen species, learning

## Abstract

The ATP synthase is an essential multifunctional enzyme complex of mitochondria that produces most of cellular ATP, shapes the structure of the inner membrane into cristae and regulates the signals that control cell fate or demise. The ATPase Inhibitory Factor 1 (IF1) functions *in vivo* as a physiological regulator of the ATP synthase and thereby controls mitochondrial structure and function, and the retrograde signaling pathways that reprogram nuclear gene expression. However, IF1 is not ubiquitously expressed in mammals, showing tissue-restricted expression in humans and mice and large expression differences between the two species in some tissues. Herein, we summarized key regulatory functions of IF1 for tissue homeostasis, with special emphasis on the deleterious effects that its genetic ablation in neurons has in learning. The development and characterization of tissue-specific mouse models with regulated expression of IF1 will be crucial to disentangle the contribution of the ATP synthase/IF1 axis in pathophysiology.

## Introduction

Mitochondria are highly dynamic organelles that play crucial metabolic functions in cellular physiology, the control of intracellular signaling and cell fate (Eisner et al., 2018; Spinelli and Haigis, 2018). The mitochondrial ATP synthase is bottleneck for energy provision because it catalyzes the synthesis of most cellular ATP by oxidative phosphorylation (OXPHOS) under aerobic conditions (Boyer, 1997; Walker, 2013). Moreover, the ATP synthase emerges as an essential hub involved in shaping the structure of mitochondrial cristae (Kühlbrandt, 2019), the permeabilization of the inner mitochondrial membrane (IMM) under physiological and pathological conditions (Mnatsakanyan and Jonas, 2020a; Carraro et al., 2020) and the control of intracellular signaling pathways (Esparza-Moltó et al., 2017).

The ATPase inhibitory factor 1 (IF1) is the physiological regulator of the ATP synthase, inhibiting both the synthetic and hydrolytic activities when it is bound to the enzyme (García-Bermúdez et al., 2015). Besides being a main regulator of mitochondrial OXPHOS, we have found that IF1 regulates mitochondrial retrograde signaling and that it is a key protein for tissue homeostasis (García-Aguilar and Cuezva, 2018). For instance, its functional relevance in synaptic transmission and learning has been recently demonstrated in mouse models of loss- and gain-of-function of IF1 in neurons (Esparza-Moltó et al., 2021). In this review, we address the role of the ATP synthase/IF1 axis in cellular physiology, highlighting its tissue specificity.

## The ATP Synthase is Crucial in OXPHOS, Cristae Structure and as Signaling Hub

**FIGURE 1:**
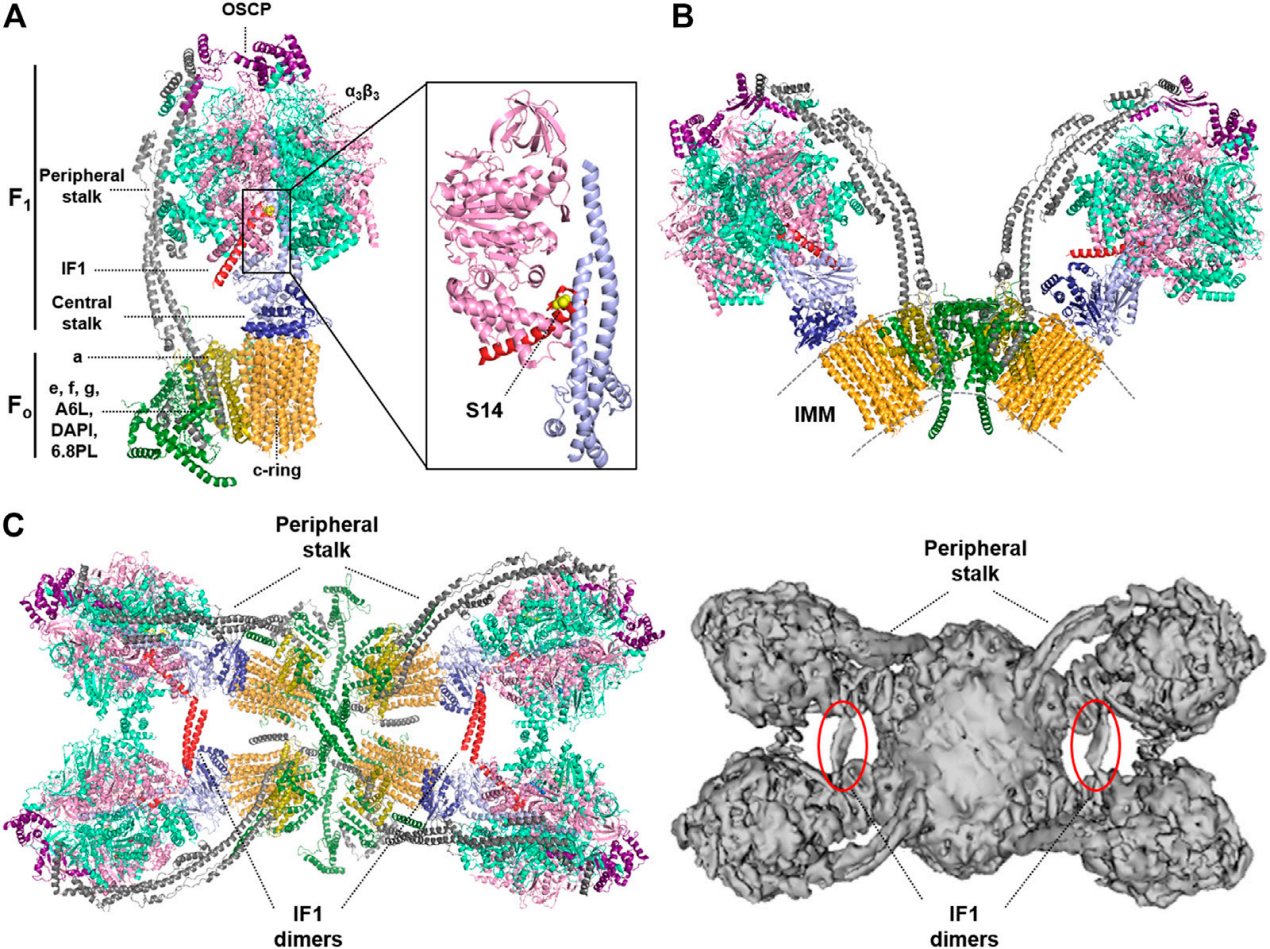
Structure of the monomer, dimer and tetramer of the mammalian ATP synthase. **(A)** Structure of monomeric bovine ATP synthase bound to the inhibitory N-terminal fragment of IF1. The soluble F_1_-ATPase domain is composed by the α3β3 subassembly (light green/pink) and the central stalk (γ subunit, light blue, and δ, ε subunits, dark blue), while the F_o_ domain is formed by a ring of 8c subunits (orange), and subunit a (dark yellow). These two domains are linked by a peripheral stalk, made up of subunits b, d, F6 (grey) and oligomycin sensitivity-conferring protein (OSCP; purple). Additional supernumerary subunits have been described in the F_o_ domain; e, f, g, A6L, diabetes-associated protein in insulin-sensitive tissues (DAPIT) and the 6.8-kDa proteolipid (6.8PL) (dark green). Inset, the interaction between the N-terminal inhibitory fragment of IF1 (red) and subunits β (pink) and γ (light blue) is shown. The position of S14 in the human and mouse inhibitory peptide is highlighted in yellow. The Ala14 (Ser14 in human and mouse IF1) is shown in yellow. Molecular reconstruction from PDB: 6ZPO. **(B)** Structure of the bovine ATP synthase dimer. The supernumerary subunits of the enzyme (DAPIT, e, f, g, A6L and 6.8PL) are involved in the dimerization of the enzyme. IMM, inner mitochondrial membrane. Molecular reconstruction from PDB: 7AJD. **(C)** Cryo-EM structures of porcine (left) and ovine (right) ATP synthase tetramers viewed from the matrix side. IF1 dimers (red or circled in red) bind two adjacent dimers of the ATP synthase promoting the formation of tetrameric or higher-order oligomers. Molecular reconstruction from PDB: 6J5K (left) and EMD-0667 (right). Images created with the PyMOL Molecular Graphics System.

The ATP synthase is the rotatory engine in the IMM that catalyzes the synthesis of ATP in a process driven by the proton-motive force, which is generated by the respiratory chain (Boyer, 1997). It is a multisubunit protein complex that consists of the membrane embedded F_o_ domain, which contains the rotor and the proton channel, and the catalytic matrix-protruding F_1_ domain, which is responsible for the synthesis of ATP (Walker, 2013; Kühlbrandt, 2019) (Figure 1A). ATP synthesis is driven by the influx of protons from the intermembrane space into the matrix, that triggers the rotation of the *c*-ring in the F_o_ domain (Figure 1A). A central stalk transfers the torque to the barrel of α_3_β_3_ subunits of the F_1_ domain, inducing the conformational changes that drive ATP synthesis (Srivastava et al., 2018; Murphy et al., 2019) (Figure 1A). A peripheral stalk acts as a stator, to prevent the unproductive rotation of the α_3_β_3_ subunits in the F_1_ domain (Hahn et al., 2018) (Figure 1A). The enzyme is assembled in a stepwise process (He et al., 2018). The F_1_ domain and the *c*-ring are assembled independently and are subsequently associated with the peripheral stalk and supernumerary subunits of the enzyme (e, f, g, 6.8pl and DAPIT). The assembly of both domains is assisted by different assembly factors (Li et al., 2017; Carroll et al., 2021). However, the assembly process is not fully understood, and different pathways have been proposed (He et al., 2020).

Mitochondrial ATP synthases form dimers in lipid bilayers, which in turn assemble into short ribbons or long rows in the IMM (Kühlbrandt, 2019; Wittig and Schägger, 2008) (Figure 1B). The supernumerary subunits of the enzyme, which are found when the complex is isolated in the presence of phospholipids, impose a local curvature on the membrane promoting its local bending (Walker, 2013; Hahn et al., 2016; Guo et al., 2017), and are involved in dimerization and oligomerization of the ATP synthase, and therefore in cristae formation (Figure 1B). Supernumerary subunits mediate different interactions between ATP synthases within a dimer and between two dimers, stabilizing its tetrameric structure as recently shown in cryo-EM studies of mammalian ATP synthases (Gu et al., 2019; Pinke et al., 2020; Spikes et al., 2020) (Figure 1C). Moreover, dimers of the enzyme are also brought together into tetramers or higher-order oligomers by protein-protein contacts between the F_1_ domain of adjacent monomers mediated by IF1 (Wittig and Schägger, 2008; Gu et al., 2019; Pinke et al., 2020) (Figure 1C). IF1 binds to the ATP synthase as a dimer with the two inhibitory domains facing opposite sites, thereby interacting with two adjacent F_1_ domains simultaneously (Cabezón et al., 2000; Gu et al., 2019; Pinke et al., 2020). However, IF1 cannot link together both F_1_ domains of the same dimer in yeast and mammals because they are too far apart (Kühlbrandt, 2019). Remarkably, this is not the case in the ATP synthase from ciliates, in which a specific subunit anchors the IF1 dimer to the membrane (Flygaard et al., 2020), or from *Toxoplasma gondii*, in which the dimers have an unusual architecture with the peripheral stalks offset and form hexamers (Mühleip et al., 2021). Interestingly, the angle between the rotatory axes in mammalian V-shaped dimers is dynamic (ranging from 76 to 95^o^ or from 80 to 90° in the bovine and ovine enzymes, respectively), indicating that the interactions between both monomers within a dimer are also dynamic (Pinke et al., 2020; Spikes et al., 2020). This dynamism may be necessary to accommodate the conformational changes that occur in the F_1_ domain during catalysis, and may also allow the enzyme to operate and/or contribute to the continuous changes in cristae architecture (Hackenbrock, 1966; Hackenbrock, 1968; Spikes et al., 2020). In any case, IF1 clearly contributes to the organization of the ATP synthase, since its overexpression in cells (Campanella et al., 2008) or *in vivo* (Santacatterina et al., 2016; Esparza-Moltó et al., 2021) increase the oligomeric assemblies of the enzyme. Moreover, genetic ablation of IF1 in neurons reduces the content of dimers and oligomers of the ATP synthase in brain mitochondria (Esparza-Moltó et al., 2021). Interestingly, IF1, by promoting the oligomerization of the ATP synthase (Faccenda et al., 2013) and the stabilization of OPA1 (Faccenda et al., 2017), has been reported to preserve cristae structure upon toxic insults and thus protect cells from apoptotic death, in agreement with previus findings (Formentini et al., 2012).

Although recently questioned (He et al., 2017; Carroll et al., 2019), increasing evidence strongly supports that the ATP synthase forms the permeability transition pore (PTP), or at least significantly contributes to it (Mnatsakanyan et al., 2019; Urbani et al., 2019; Carraro et al., 2020; Carrer et al., 2021). The PTP is the mitochondrial megachannel whose prolonged opening permeabilizes the IMM to small solutes and commits cells to death (Carraro et al., 2020). However, the PTP also undergoes physiological transient openings known as “flickering” that are key for buffering matrix Ca^2+^, regulating the efficiency of OXPHOS and the production of mitochondrial reactive oxygen species (mtROS) (Mnatsakanyan and Jonas, 2020a; Carraro et al., 2020). Therefore, the ATP synthase emerges as a key player in OXPHOS, cristae structure, the execution of cell death and signaling.

## IF1 is the Physiological Regulator of the ATP Synthase

IF1 is a structurally disordered protein that binds to the catalytic interface in the F_1_ domain (Gledhill et al., 2007). During the binding process, the disordered region of IF1 interacts with the most open of the three catalytic sites and becomes α-helical as it establishes more interactions with the enzyme (Bason et al., 2014) and once bound, blocks the rotatory catalysis of the complex (Figure 1). For many years now, IF1 has been considered an inhibitor only of the ATP hydrolytic activity of the enzyme, the so-called unidirectional inhibitor of the enzyme (Walker, 2013). In this situation, IF1 prevents reverse functioning of the enzyme to maintain the mitochondrial membrane potential (ΔΨm) when the organelles become de-energized in conditions of hypoxia (Campanella et al., 2008; Walker, 1994). This notion is largely based on *in vitro* findings showing that IF1 readily inhibits ATP hydrolysis by the isolated enzyme (Walker, 2013; Gledhill et al., 2007; Bason et al., 2014; Walker, 1994; Cabezon et al., 2000). However, more recent findings indicate that IF1 also inhibits the forward ATP synthetic activity of the enzyme, as revealed by a reduction in the oligomycin-sensitive respiratory rate in cells overexpressing IF1 (Formentini et al., 2012; Sánchez-Cenizo et al., 2010; Kahancová et al., 2018; Kahancová et al., 2020) or in the oligomycin-sensitive ATP synthesis rate assessed in isolated mitochondria and in permeabilized cells (García-Bermúdez et al., 2015; Nuevo-Tapioles et al., 2020). Moreover, the ATP synthetic activity was also significantly inhibited in isolated mitochondria of different tissues in transgenic mice overexpressing IF1 *in vivo* (Santacatterina et al., 2016; Formentini et al., 2014; Formentini et al., 2017; Sánchez-González et al., 2020). In addition, the IF1-mediated inhibition of the ATP synthetic activity of the enzyme was also traced by the activation of glycolysis through signaling pathways sensing the reduction in cellular ATP availability, recapitulating the effect of oligomycin (Santacatterina et al., 2016; Formentini et al., 2012; Sánchez-Cenizo et al., 2010; Formentini et al., 2014; Formentini et al., 2017; Sánchez-Aragó et al., 2013a). Independent findings indicated that IF1 inhibits the translocation of protons mediated by the ATP synthase in submitochondrial particles or in reconstituted liposomes when operating either in the synthetic or hydrolytic modes (Zanotti et al., 2009). More direct evidence was provided recently using genetic models for the IF1-mediated regulation of the ATP synthase (Esparza-Moltó et al., 2021). Indeed, ablation of IF1 in mouse neurons increases both ATP hydrolase and synthetic activities of the ATP synthase in isolated mitochondria, whereas its overexpression increases the fraction of IF1 bound to the enzyme and reduces both ATP synthase and hydrolase activities of the enzyme (Esparza-Moltó et al., 2021). These *in vivo* and cellular findings are in full agreement with the recent cryo-EM structures of the tetrameric ATP synthase purified from porcine or ovine heart mitochondria, that reveal under physiological conditions that IF1 is bound and inhibits the enzyme (Figure 1C) (Gu et al., 2019; Pinke et al., 2020).

## IF1 is Expressed in a Tissue-Specific Manner and its Inhibitory Activity is Regulated by Phosphorylation

IF1 is highly overexpressed in most prevalent human carcinomas and contributes to the reprogramming of metabolism towards an enhanced glycolytic flux in cancer and non-cancer cells (Sánchez-Cenizo et al., 2010; Sánchez-Aragó et al., 2013a; Esparza-Moltó et al., 2017; Sánchez-González et al., 2020). Cancer cells and undifferentiated cells with high proliferation rates show metabolic similarities (Zhang et al., 2012). Interestingly, IF1 expression is also increased in stem cells when compared to some differentiated cells (Sánchez-Aragó et al., 2013b). Indeed, downregulation of IF1 in adult human mesenchymal stem cells is necessary for their osteogenic differentiation (Sánchez-Aragó et al., 2013b). Along the same line, somatic cell reprograming is associated with the upregulation of protein levels of IF1 (Prieto et al., 2018). Hence, IF1 may be a marker for proliferation and stemness, and may play a role in shaping the metabolic profile of these cells by restraining OXPHOS and favoring glycolysis.

**FIGURE 2:**
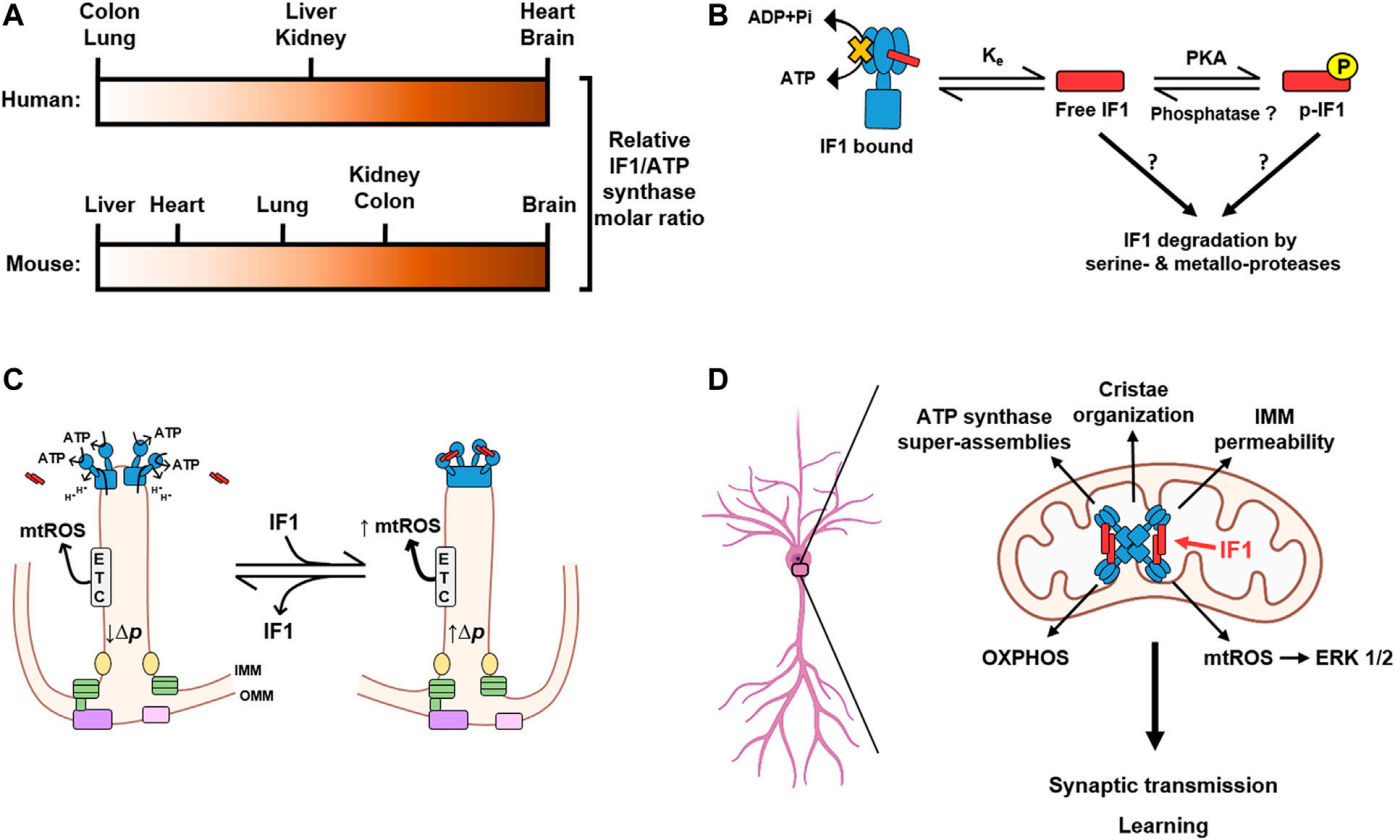
IF1 is tissue-specifically expressed and plays a central role in neuronal function and learning. **(A)** The relative molar ratio between IF1 and the ATP synthase varies in different tissues and between human (above) and mouse (below). Negligible IF1 expression, white; Highest ratio, dark brown. **(B)** Interaction of IF1 with the ATP synthase is a reversible process mainly dependent on the mitochondrial content of dephosphorylated and free IF1. IF1 is phosphorylated by a mitochondrial protein kinase A (PKA)-like activity, thus reducing the amount of free IF1 that can bind to the enzyme. No phosphatases have been yet found to mediate IF1 dephosphorylation. An additional actor affecting the binding equilibrium of IF1 (Ke) might be the rapid turnover of IF1 mediated by serine- and metallo-proteases. **(C)** Cartoon illustrating the major effects of IF1 binding to ATP synthase in its activity and in generating the tetrameric structure of the enzyme to facilitate the formation of cristae rims. Binding of IF1 (red) to dimers of the ATP synthase (blue) generate tetramers of inhibited enzyme leading to increased proton-motive force (Δ*p*) in cristae and the generation of mitochondrial ROS (mtROS) by the electron transport chain (ETC). Other components that facilitate cristae structure such as the MICOS complex (green), mitochondrial dynamin-like GTPase (OPA1, yellow), the sorting and assembly machinery (SAM, purple) and translocase of the outer membrane (TOM, pink) located at the inner boundary membrane are depicted. **(D)** The expression of IF1 in hippocampal neurons promotes the formation of super-assemblies of the ATP synthase and thereby contributes to the organization of mitochondrial cristae and the permeability of the inner mitochondrial membrane (IMM). Moreover, IF1 plays a crucial role in the control of oxidative phosphorylation (OXPHOS) and signaling mediated by mitochondrial reactive oxygen species (mtROS), which activate the extracellular signal-regulated kinases (ERK) 1/2. By modulating these processes, IF1 emerges as a relevant protein for the regulation of synaptic transmission and learning. Image produced with BioRender.

In contrast, IF1 shows a cell type-specific expression pattern in normal adult tissues (Sánchez-Cenizo et al., 2010; Esparza-Moltó et al., 2019) (Figure 2A). For instance, the epithelia of human colon and lung contain low levels of IF1, while the heart, brain, kidney and liver contain considerable amount of IF1 protein (Esparza-Moltó et al., 2019). A semiquantitative approach aimed at investigating the relative content of IF1 over the ATP synthase revealed that human heart and brain have a molar excess of the inhibitor over the enzyme (Esparza-Moltó et al., 2019) (Figure 2A). Moreover, the expression pattern of IF1 in mouse tissues differs from that in human tissues. Whereas both human and mouse brain express high levels of the protein, mouse heart and liver express low levels of it (Esparza-Moltó et al., 2019) (Figure 2A). Conversely, and in contrast to its human counterpart, mouse colon expresses high levels of IF1, being in molar excess over the enzyme (Esparza-Moltó et al., 2019) (Figure 2A).

The observation that tissues with high energy demand that rely on an efficient OXPHOS system for energy provision express high levels of IF1 was a puzzling scenario. However, this energetic paradox was partially solved after showing that IF1 inhibitory activity can be abolished by the phosphorylation of S39 (S14 in the mature human and mouse IF1) (García-Bermúdez et al., 2015). And indeed, a relevant fraction of IF1 is phosphorylated in mouse tissues with a molar excess of the inhibitor protein (Esparza-Moltó et al., 2019), thus reducing the tissue content of IF1 that can bind and inhibit the activity of the enzyme. However, another relevant fraction of IF1 is dephosphorylated and co-migrates with different assemblies of the ATP synthase (Esparza-Moltó et al., 2019), indicating inhibition of its activity (Santacatterina et al., 2016). These findings suggest the existence of two pools of the ATP synthase under basal physiological conditions: one actively producing ATP and one inhibited by IF1, in agreement with similar observations in mouse heart in response to *in vivo* stimulation of β-adrenergic signaling (García-Bermúdez et al., 2015; García-Bermúdez and Cuezva, 2016) and cryo-EM structures of mammalian ATP synthase (Figure 1C) (Gu et al., 2019; Pinke et al., 2020). Importantly, modulating the dose of IF1, either by its knock-out or overexpression, affects the fraction of IF1 bound to the ATP synthase paralleling the reduction of both the ATP hydrolase and synthase activities of the enzyme (Esparza-Moltó et al., 2021). Hence, the interaction of IF1 with the ATP synthase depends on the mitochondrial content of the inhibitor and therefore it is controlled by the mass action ratio (Figure 2B). The phosphorylation of IF1 provides an additional level for the regulation of the ATP synthase activity by affecting the fraction of the inhibitor that can bind to the enzyme (Figure 2B).

The existence of a bulk of ATP synthase inhibited by IF1 may be relevant for the fine-tuning of ATP provision by OXPHOS with the cellular energy demand (García-Bermúdez et al., 2015). Indeed, β-adrenergic stimulation of mice, a condition that mimics a situation of high energy demand, triggers the phosphorylation of heart IF1 and an increase in the production of ATP in mitochondria (García-Bermúdez et al., 2015). However, the proteins that mediate the phosphorylation of IF1 are ill-defined. IF1 is phosphorylated by a cAMP-dependent protein kinase A like activity within mitochondria (García-Bermúdez et al., 2015) (Figure 2B). A soluble adenylate cyclase (sAC) is the source of cAMP in mitochondria (Acin-Perez et al., 2009) and it is activated upon mitochondrial uptake of Ca^2+^ (Di Benedetto et al., 2013). Since Ca^2+^ triggers the contraction of muscle fibers and is sequestered in mitochondria (Rizzuto et al., 2012), the Ca^2+^/sAC/IF1 axis provides a mechanism coupling the increased energy demand imposed by β-adrenergic stimulation of the heart with a higher ATP production in mitochondria. In other words, phosphorylation of IF1 relieves the brake on a fraction of ATP synthase to supply more ATP when it is needed. On the other hand, IF1 is dephosphorylated in cells under hypoxic conditions or when progressing through the glycolytic phases of the cell cycle (García-Bermúdez et al., 2015). No phosphatases have been yet identified regulating IF1 phosphorylation status. Hence, it is reasonable to suggest that its rapid turnover (Sánchez-Aragó et al., 2013a; Sánchez-Aragó et al., 2013b), when compared to other subunits of the ATP synthase (García-Aguilar et al., 2019), could participate as an additional mechanism controling the mitochondrial content of phospho- and dephospho-IF1 (Figure 2B). Interestingly, the expression and phosphorylation of IF1, which is present in mitochondria from pancreatic β-cells, regulates glucose-stimulated insulin secretion by controlling ATP production in mitochondria and thus the ATP/ADP ratio (Kahancová et al., 2018; Kahancová et al., 2020), highlighting its relevance in metabolic regulation.

## Two Pools of ATP Synthase, Cristae Heterogeneity and mtROS Signaling

Moreover, the existence of the two pools of the ATP synthase could contribute to the heterogeneity in ΔΨm (Wolf et al., 2019) and ΔpH (Rieger et al., 2021) that has been recently reported in mitochondrial cristae by high spatial resolution microscopy. The active and inactive fractions of ATP synthase could be in functionally independent cristae, or even in different regions within the same cristae, thereby contributing to the differences in ΔΨm and ΔpH that affect the overall activity of oxidative phosphorylation (Figure 2C). Therefore, IF1, by favoring the formation of ATP synthase tetramers which promote cristae formation and are inhibited for the handling of ATP, may increase the number of cristae in which ATP synthesis is slowed down and the proton-motive force (Δ*p)* is increased (Figure 2C). In this way, we propose that there are microdomains of the enzyme in cristae containing active and inactive ATP synthases, and that the latter domains are preferentially located at cristae rims in order to stabilize and facilitate the generation of the infolds of the IMM (Figure 2C). The distribution of IF1-inhibited ATP synthase at cristae rims differs from the ETC distribution at the flat region of cristae, and generates mtROS as a function of the fraction of the ATP synthase that is inhibited by IF1 (Figure 2C). The asymmetric distribution of ETC and IF1-inhibited ATP synthase in cristae results in a heterogeneous distribution of Δ*p* along the cristae or in different cristae (Figure 2C). However, these distributions are highly dynamic, changing in the time scale of seconds or less and depending on a very large number of factors. In this way, the “poised” cristae may be a reservoir of enzyme ready to respond to an increase in energy demand or operate as signaling “modules” by increasing ROS production.

The IF1-mediated inhibition of the ATP synthase promotes an increase in the proton-motive force and in the production of mtROS, since ΔΨm and mtROS production concurrently increase with higher IF1 dose in neurons (Esparza-Moltó et al., 2021). The increased mtROS production rate can be explained by increased reverse electron transfer (RET) to complex I, since ΔΨm controls the level of RET (Robb et al., 2018). RET is a relevant pathway in ischemia-reperfusion injury due to the aberrant production of mtROS (Chouchani et al., 2014), but it is also involved in the polarization of macrophages (Mills et al., 2016) and promotes mitochondrial function and longevity in fly models of Parkinson disease and aging (Scialò et al., 2016). Indeed, mtROS regulate the activity of kinases and transcription factors involved in the control of nuclear and cellular responses necessary for the adaptation to changing cues (Holmström and Finkel, 2014). In this regard, IF1 emerges as a key regulator of retrograde signaling pathways that control nuclear gene expression programs (Esparza-Moltó et al., 2017). The partial arrest of OXPHOS by IF1 overexpression in different tissues *in vivo* represents a mild stress in mitochondrial function, but results in the activation of long-lasting metabolic and molecular cytoprotective mechanisms that allow the cells to withstand subsequent insults (Formentini et al., 2014; Santacatterina et al., 2016; Formentini et al., 2017), that is, IF1 signals mitohormetic processes (Esparza-Moltó et al., 2017). Interestingly, genetic and metabolic studies targeting the ATP synthase reveal that it regulates lifespan, as its silencing (Dillin et al., 2002; Hansen et al., 2005; Sun et al., 2014) or the inhibition of its activity (Chin et al., 2014; Fu et al., 2015) promote longevity in different model organisms by activating mitohormetic signaling. Although genetic regulation of IF1 dose in neurons revealed no relevant effect in the life span of mice (Esparza-Moltó et al., 2021), it was remarkable to observe that transgenic mice overexpressing IF1 had increased exploratory activity, better motor coordination and long-term memory than wild type and mice devoid of IF1 in neurons (Esparza-Moltó et al., 2021).

## Detrimental Effects of IF1 Overexpression in Mouse Tissues That Contain Low Levels of IF1

Nevertheless, the biological significance of the IF1-mediated inhibition of the ATP synthase is more intricate and emphasizes its tissue- and species-specific role in mitochondrial functions. In fact, the overexpression of IF1 in mouse tissues that naturally express low levels of the protein has detrimental effects for the animal. For instance, mice that overexpress a constitutively active mutant of IF1 in the liver are more prone to hepatocarcinogenesis upon diethyl-nitrosamine administration, stressing the pro-oncogenic role of IF1 in liver cancer progression (Santacatterina et al., 2016; Formentini et al., 2012; Song et al., 2014). Moreover, the partial arrest of OXPHOS triggered by IF1 overexpression in mouse skeletal muscle alters whole-body lipid homeostasis and results in metabolic syndrome (Sánchez-González et al., 2020). In mouse heart, loss of LRPPRC (leucine-rich pentatricopeptide repeat containing protein), which recapitulates a rare form of Leigh syndrome, causes a dramatic increase in the content of IF1 protein (Mourier et al., 2014). This occurs at the post-transcriptional level, since LRPPRC binds and represses the translation of IF1 mRNA in mouse heart (Esparza-Moltó et al., 2019; Esparza-Moltó and Cuezva, 2020). IF1 upregulation in LRPPRC-knockout mice in the heart results in a progressive lethal cardiomyopathy, caused by an alteration in the assembly and oligomerization of the ATP synthase that leads to a bioenergetic impairment (Mourier et al., 2014). Consistent with these findings, it has been reported that IF1 could contribute to cardiac damage in a mouse model of cardiac hypertrophy (Yang et al., 2017). Remarkably, and in sharp contrast to mouse heart, IF1 is highly expressed in human heart under normal conditions, emphasizing large differences in IF1 function in this organ between both species (Esparza-Moltó et al., 2019; Rouslin, 1987) (Figure 2A). It remains to be deciphered how IF1 expression in mouse heart promotes cardiac damage. We suggest that the availability of genetically modified IF1 mice could contribute in this regard (Formentini et al., 2014; Esparza-Moltó et al., 2021).

Overall, we suggest that the regulation of the ATP synthase by IF1 has tissue-specific functional relevance, and that this relevance is imposed by the restricted expression pattern of IF1 (Esparza-Moltó et al., 2019) (Figure 2A). This is consistent with the proteomic and functional specialization of mitochondria in different tissues thanks to the fine and specific adjustment of their regulatory mechanisms (Pagliarini et al., 2008). However, little is known about the physiological role of IF1 *in vivo* in cell types that express high content of the inhibitor, such as neurons from both the human and mouse.

## The ATP Synthase/IF1 Axis is Key for the Regulation of Neuronal Function

Neurons are highly specialized cells that consume around 75% of the energy produced in the brain (Magistretti and Allaman, 2015). Most of this energy budget accounts for synaptic processes, including the replenishment of pre-synaptic vesicles with neurotransmitters and the maintenance of the resting membrane potential in post-synaptic terminals (Magistretti and Allaman, 2015). The pre-synaptic vesicle cycle is fueled by both glycolysis and OXPHOS, which are stimulated by the electrical activity (Rangaraju et al., 2014). However, little is known about the energy supply in post-synaptic terminals. Recent findings show that mitochondria spatially confined in dendritic spines fuel local protein translation during synaptic plasticity (Rangaraju et al., 2019). While neurons can consume glucose, they obtain most ATP through the oxidation of astrocytic-derived lactate, especially during periods of high synaptic activity, as proposed in the astrocytic-neuron lactate shuttle (Magistretti and Allaman, 2018). Hence, it appears paradoxical from the energetic viewpoint that most IF1 expression in the brain is restricted to neurons, while the more glycolytic astrocytes contain negligible amount of the inhibitor of the ATP synthase (Esparza-Moltó et al., 2019).

Notably, the genetic ablation of IF1 in forebrain neurons impairs learning in mice, while its overexpression promotes long-term memory, indicating a key role for IF1 in the regulation of neuronal function and cognition (Esparza-Moltó et al., 2021). IF1-knockout mice in neurons show reduced content of oligomeric assemblies of the ATP synthase in forebrain mitochondria and an altered cristae structure, while IF1 overexpression promotes the oligomerization of the enzyme and a more organized cristae (Esparza-Moltó et al., 2021). In fact, it has been reported that zebrafish and mouse models lacking IF1 show increased cell death in the central nervous system and in the retina, and thereby have a mild visual impairment (Martín-Jiménez et al., 2018). This is associated with reduced OPA1 expression that likely affects cristae organization (Martín-Jiménez et al., 2018). Interestingly, a defect in the expression of subunit *k* of the ATP synthase (also known as DAPIT), caused by a splice mutation found in Leigh syndrome patients also reduces the content of ATP synthase dimers (Barca et al., 2018). Mitochondria from fibroblasts collected from these patients show altered cristae organization (Barca et al., 2018), thus supporting the role of ATP synthase oligomers in cristae architecture and mitochondrial function. Moreover, DAPIT has been recently identified as a susceptibility gene for schizophrenia and its deficiency affects mitochondrial respiration, neuronal development and mouse behavior (Wang et al., 2021). Hence, it is conceivable that IF1 might also play a relevant role in neuronal function by regulating the assembly of the ATP synthase and cristae organization (Figure 2D).

The expression of other subunits of the ATP synthase is also altered in different neurological diseases. For instance, OSCP is downregulated in Alzheimer disease patients and mouse models (Beck et al., 2016). The expression of *β*-F1-ATPase, the catalytic subunit of the ATP synthase, is reduced in a mouse model of Parkinson disease (Chen et al., 2019). In both cases, there is an altered stoichiometry between the F_1_ and F_o_ domains of the enzyme that destabilizes the complex (Beck et al., 2016; Chen et al., 2019), favoring the dissociation of the F_1_ domain (Mnatsakanyan and Jonas, 2020b). Moreover, cyclophilin D -a protein that binds to the ATP synthase and induces the opening of the PTP (Carraro et al., 2020)- promotes the selective loss of OSCP subunit in aged mice and in models of Alzheimer disease (Gauba et al., 2017; Gauba et al., 2019). This way, cyclophilin D mediates the dysfunction of the ATP synthase in these conditions.

It has been described that the ATP synthase harbors the uncoupling channel that contributes to the PTP in the membrane-embedded *c*-ring (Bonora et al., 2013; Alavian et al., 2014; Mnatsakanyan et al., 2019). An excessive opening of the PTP has been reported in neurons from mouse models of Parkinson disease (Chen et al., 2019) and fragile X syndrome (Licznerski et al., 2020). It is caused by the dissociation of the F_1_ domain of the ATP synthase and an increase in free *c*-rings, which augment mitochondrial proton leak (Licznerski et al., 2020). The excessive leak affects neuronal metabolism and synaptic function and mediates an autistic-like behavior in mouse models of fragile X syndrome (Licznerski et al., 2020). Interestingly, IF1 expression is downregulated in the brain of the same fragile X syndrome mouse model (Xu et al., 2018). We have found that neurons from IF1-knockout mice show an increased mitochondrial proton leak that promotes less efficient OXPHOS (Esparza-Moltó et al., 2021). Conversely, IF1 overexpression in neurons promotes better organized cristae structure in mitochondria with less proton leak, which might contribute to more efficient energy provision, increased size of dendritic spines and higher synaptic transmission (Esparza-Moltó et al., 2021). Therefore, IF1, by contributing to the oligomerization of the ATP synthase, could regulate the permeability of the IMM and thereby plays a relevant role in neuronal metabolism and learning (Figure 2D).

Finally, we have recently shown that IF1 also regulates neuronal function through the control of mtROS production in neurons (Esparza-Moltó et al., 2021) (Figure 2D). mtROS promote neuronal activation in transgenic mice overexpressing IF1 by signaling through the activation of extracellular signal-regulated kinases (ERK) 1/2 (Esparza-Moltó et al., 2021). ERK 1/2 is a crucial hub whose signaling contributes to the induction of synaptic plasticity and learning (Sweatt, 2001). ROS activate ERK 1/2 (Kanterewicz et al., 1998), likely by modulating the activity of upstream kinases and phosphatases, and this way mtROS regulate signaling pathways involved in synaptic function (Oswald et al., 2018).

Neurons and glial cells work together in the brain forming an intricate association that is necessary for proper neuronal and higher-order brain functions (Magistretti and Allaman, 2018). In this regard, the physiological role of mtROS depends on which cell type they are produced in (Jimenez-Blasco et al., 2020; Vicente-Gutierrez et al., 2021). Astrocytic mtROS are necessary for neuronal survival, because they stimulate the production of lactate in astrocytes, which is delivered to neurons via the lactate shuttle and supports neuronal function (Jimenez-Blasco et al., 2020). Indeed, reducing mtROS production in astrocytes by the specific activation of type-1 cannabinoid receptors present in astroglial mitochondria impairs neuronal function and social behavior in mice (Jimenez-Blasco et al., 2020). However, quenching mtROS production in neurons by the overexpression of a mitochondrial targeted catalase does not affect behavior under basal conditions (Vicente-Gutierrez et al., 2021). Nevertheless, we cannot rule out a role for neuronal mtROS. They have been shown to regulate excitatory (Fu et al., 2017; Esparza-Moltó et al., 2021) and inhibitory (Accardi et al., 2014) neurotransmission and learning (Esparza-Moltó et al., 2021). Definitively, where and how ROS are being produced in mitochondria is relevant for their functional outcome (Scialò et al., 2016), especially in responses as complex as learning. The site and mechanism of mtROS production might affect which signaling molecules are modified and therefore the downstream responses that are induced.

## Conclusion

The ATP synthase is a crucial hub in mitochondria that integrates OXPHOS, the organization of mitochondrial cristae, the permeabilization of the IMM, and the regulation of intracellular signaling. Using both pharmacological and genetic approaches, we have shown that IF1 functions *in vivo* as a physiological inhibitor of the ATP synthase. The IF1-mediated inhibition of the ATP synthase is a physiologically relevant mechanism to adapt ATP production by OXPHOS with the cellular energy demand, control ΔΨm and mitochondrial retrograde signaling mediated at least by mtROS. Moreover, it regulates the oligomerization of the ATP synthase and the organization of mitochondrial cristae. However, very little is known about the precise regulation of the ATP synthase by IF1. In this regard, it will be interesting to address whether the IF1-inhibited fraction of ATP synthase promotes localized mitochondrial hyperpolarization and mtROS production by the electron transport chain in functionally independent cristae, or even within different regions of the same cristae, rather than by overall increase of ΔΨm in the whole mitochondrion.

Moreover, the biological relevance of the ATP synthase/IF1 axis is far from being fully understood. The tissue-restricted expression of IF1 may explain why its overexpression either *in vitro* or *in vivo* yields different outcomes depending on the cellular type. In neurons, which show ample expression of the inhibitor in both humans and mice, IF1 is a crucial protein regulating synaptic transmission and memory as well as for protection from excitotoxic insults (Formentini et al., 2014). Hence, the ATP synthase/IF1 axis offers a valuable therapeutic target to treat cognitive deficits associated with neurodegenerative and age-associated conditions (Goldberg et al., 2018). However, the expression of IF1 differs between the human and mouse in other tissues, such as colon and heart. The development of tissue-specific mouse models lacking or overexpressing IF1 will be invaluable to delineate the physiological and adaptive processes that are modulated by the ATP synthase/IF1 axis to unveil their contribution to the control of cellular function and pathophysiology.
